# Monitoring of Copper in Wilson Disease

**DOI:** 10.3390/diagnostics13111830

**Published:** 2023-05-23

**Authors:** Grażyna Gromadzka, Marta Grycan, Adam M. Przybyłkowski

**Affiliations:** 1 Medical Faculty, Collegium Medicum, Cardinal Stefan Wyszyński University in Warsaw, Wóycickiego Street 1/3, 01-938 Warsaw, Poland; 2 Students Research Club, Maria Sklodowska-Curie Medical Academy, 03-411 Warsaw, Poland; 3 Department of Gastroenterology and Internal Medicine, Medical University of Warsaw, 02-091 Warsaw, Poland

**Keywords:** Wilson disease, diagnostics, copper, ceruloplasmin, radioactive copper, non-ceruloplasmin copper, free copper, relative exchangable copper

## Abstract

(1) Introduction: Wilson’s disease (WND) is an autosomal recessive disorder of copper (Cu) metabolism. Many tools are available to diagnose and monitor the clinical course of WND. Laboratory tests to determine disorders of Cu metabolism are of significant diagnostic importance. (2) Methods: A systematic review of the literature in the PubMed, Science Direct, and Wiley Online Library databases was conducted. (Results): For many years, Cu metabolism in WND was assessed with serum ceruloplasmin (CP) concentration, radioactive Cu test, total serum Cu concentration, urinary copper excretion, and Cu content in the liver. The results of these studies are not always unambiguous and easy to interpret. New methods have been developed to calculate non-CP Cu (NCC) directly. New parameters, such as relative Cu exchange (REC), reflecting the ratio of CuEXC to total serum Cu, as well as relative Cu exchange (REC), reflecting the ratio of CuEXC to total serum Cu, have been shown to be an accurate tool for the diagnosis of WND. Recently, a direct and fast LC-ICP-MS method for the study of CuEXC was presented. A new method to assess Cu metabolism during treatment with ALXN1840 (bis-choline tetrathiomolybdate [TTM]) has been developed. The assay enables the bioanalysis of CP and different types of Cu, including CP-Cu, direct NCC (dNCC), and labile bound copper (LBC) in human plasma. Conclusions: A few diagnostic and monitoring tools are available for patients with WND. While many patients are diagnosed and adequately assessed with currently available methods, diagnosis and monitoring is a real challenge in a group of patients who are stuck with borderline results, ambiguous genetic findings, and unclear clinical phenotypes. Technological progress and the characterization of new diagnostic parameters, including those related to Cu metabolism, may provide confidence in the more accurate diagnosis of WND in the future.

## 1. Introduction

Wilson’s disease (WND) [OMIM #277900] is an autosomal recessive disorder of copper metabolism. The worldwide prevalence of Wilson’s disease is estimated at about 30 in 1 million, with a carrier rate of 1 in 90, with a higher prevalence is found in Sardinia, where about 10–12 new cases are identified per year (https://www.omim.org/entry/277900, accessed on 5 April 2023). In the 1940s, Cummings documented that WND is associated with excessive copper accumulation in the organs [1]. Subsequently, it was found that patients with WND had reduced serum ceruloplasmin (CP) activity [2], and later in 1993, the WND gene was identified WND [3,4]. The copper-transporting adenosine triphosphatase P-type 7B (ATPase7B), encoded by the *ATP7B* gene, is involved in the excretion of excess copper from cells, mainly hepatocytes, into the bile. Its deficiency or functional impairment results in copper accumulation within the liver and other organs. Copper depositions in the brain, kidney, cornea, and other organs results in the onset of clinical symptoms of WND. WND may manifest with variable symptoms that depend on the organs where copper is accumulated. Liver and neurological involvement predominates, and WND most frequently manifests in a form of hepatic, neuropsychiatric, or mixed symptoms. [5]

The aim of the paper is to present the current state of knowledge on the biochemical tests of copper metabolism in the diagnosis and monitoring of WND.

## 2. Materials and Methods

A systematic review of the literature in the PubMed, Science Direct, and Wiley Online Library databases has been conducted based on the following keywords: “copper”, “Wilson’s disease”, “copper biochemical studies”, “copper metabolism”, “biochemical diagnostics”, “copper and Wilson disease and treatment”, and combinations thereof.

The bibliography of extracted articles was analyzed. Older works are cited in order to depict the history and introduce the reader to the presented issues. Recent papers are cited to present the current knowledge on the research problem.

## 3. Results

### 3.1. Copper Metabolism in WND–Laboratory Monitoring

For many years, copper metabolism in WND was most often assessed on the basis of: serum CP concentration, serum total copper concentration, urinary copper excretion, and copper deposits in the liver [6]. None of the laboratory markers of copper accumulation is 100% meaningful and specific for WND. Below, the copper monitoring methods are discussed, and traditional and new tests are presented (their key features and major limitations are also summarized in Table 1).

#### 3.1.1. Ceruloplasmin

One consequence of the loss of functional ATPase7B is the lack of copper incorporation into apo-CP. Apo-CP is mainly synthesized in the liver (low expression of CP has also been found in the kidneys, large intestine, adrenal glands, lungs and brain) [7,8]. Apo-CP has a short half-life of approximately 5 h and no oxidase activity. The incorporation of 6–8 copper atoms into the apo-CP molecule determines the formation of a mature, fully active CP molecule (called holo-CP). It has a longer half-life (5.5 days) than apo-CP [9,10].

ATPase7B transports copper to apo-CP, and if this does not happen, apo-CP is rapidly degraded, and, therefore, patients with WND have decreased serum CP.

An important difficulty regarding the use of serum CP (different forms of CP are measured by different tests; enzyme assay mainly measures holo-CP and nephelometric assay measures both apo- and holo-CP) in the diagnosis of WND is that some patients with WND have CP within the reference range [11]. A few studies from Europe showed that approximately 15–30% of patients with WND had a serum CP concentration > 20 mg/dL [12,13,14]. Normal serum CP may be associated with *ATP7B* mutations that allow copper to be transported to the trans-Golgi network where CP is formed. Because CP is an acute phase protein, it may also be within the normal range in patients with Wilson’s disease during inflammation [12,15,16].

The CP may also rise to the normal range in patients with WND who are pregnant, taking oral contraceptives, and suffering from infections and hepatitis [17]. Approximately 50% of patients with acute decompensated liver failure have serum CP levels within established normal limits [18]. Serum CP may be low in conditions with marked loss of renal or intestinal protein, in patients with severe end-stage liver disease of any etiology, or in a course of rare neurological diseases [19]. Low CP and/or the pancytopenia have been recognized in patients with copper deficiency [20] and in patients with Menkes disease, an X-linked disorder of copper transport due to mutations in *ATP7A* [21]. Patients with aceruplasminemia are completely protein deficient for CP mutations on chromosome 3, and these patients present symptoms of iron overload (CP is ferroxidase) rather than copper accumulation [22,23].

About 85% of presymptomatic WND patients had decreased CP concentration [24]. In a study of asymptomatic children with WND, serum CP was significantly lower in patients with WND than in healthy children, and only a small percentage (2.3%) of asymptomatic children with WND had normal (>20 mg/dL) serum CP levels [25,26]. Approximately 20% of heterozygotes had reduced serum CP [27].

There are different ranges of blood CP values that are considered normal, depending on the laboratory and the cut-off values of serum CP concentrations for the diagnosis of WND range from 11.5 mg/dL to 20 mg/dL [28,29,30,31].

In a recent systematic review, the authors showed that a threshold concentration of 0.20 g/L had a sensitivity of 77.1–99.0% and a specificity of 55.9–82.8%, while values < 0.1 g/L are associated with a sensitivity of 65.0–78.9% and a specificity of 96.6–100% [32]. However, it has been reported that in patients with acute liver failure, a serum CP cut-off of 20 mg/dL provided only 21% diagnostic sensitivity and 84% specificity for WND [33,34]. In one study, a CP threshold of <0.14 g/L resulted in a better specificity of 98.1% than the specificity of 84.2% given by a cut-off value of 0.20 g/L with a similar sensitivity. A serum CP cut-off value of 16 mg/dL resulted in the highest diagnostic accuracy of WND in patients with acute hepatic failure [35]. In another study, Receiver Operating Characteristic (ROC) Curve Analysis showed that the optimal serum CP cut-off value is 16.85 mg/dL, with a sensitivity of 95.9% and a specificity of 93.6% for the diagnosis of WND in children. Using the conventional serum CP cut-off value of 20 mg/dL, the sensitivity is 98.1% and the specificity is 86.5% [11,36]. This result was consistent with the results of other Chinese studies on adult patients with WND, indicating that the serum CP threshold of 16.8 mg/dL may be more accurate and favor higher accuracy in the diagnosis of WND in both children and adults [37]. The ROC curve analysis indicated that the cutoff value of serum CP level at 16.8 mg/dL may provide the highest accuracy for diagnosis of WND. The cutoff value of serum CP level of 16.85 mg/dL provided the highest Area Under the Curve (AUC) value of 0.990 (95% confidence interval (CI), 0.985–0.995), with a sensitivity of 95.9% and specificity of 93.6%. The positive and negative predictive values of serum CP level of 16.85 mg/dL were 89.7% and 97.5%, respectively.

A standardized reference method for ceruloplasmin is not yet available. Two different methods are commonly used to determine the concentration of CP in serum: oxidase or nephelometric. The results of serum CP concentration determination with these methods are treated as equivalent, and similar reference ranges have been adopted for both methods (25–45 mg/dL for the oxidase method and 24.5–39.9 mg/L [range suggested by the manufacturer of the reagents used in research] to nephelometry). However, the principle of determination in these methods is different. The oxidase method evaluates the concentration of CP on the basis of enzymatic activity, and the nephelometric method directly determines the protein concentration in the blood serum based on the reactivity of the protein with a monoclonal antibody that is directed against apo- and holo-CP [38]. Apo-CP is an inactive protein that breaks down after a few minutes, but still contributes to the total CP concentration measured by this method. Walshe demonstrated that CP values obtained immunologically were higher than CP oxidase values [39].

In a comparative study using an enzymatic test and an immunologic assay for CP measurement, the enzymatic approach based on serum CP oxidase activity resulted to obtain a better cut-off point for predicting WND [40].

Walshe et al. consider the possibility of the presence of low- and high-molecular substances in the serum that inhibit the oxidase activity of CP. One of them is ascorbic acid. Other authors also speculate that serum substances may reduce CP activity by up to 25% and postulate the addition of purified CP during the oxidase determination in order to equalize the activity [41].

The ceruloplasmin concentration/activity in patients with WND may depend on the type of biological material used. 

Although most published CP enzyme assays involve the use of serum samples, it has been suggested that plasma samples derived from ethylenediamine tetraacetic acid (EDTA)-treated blood are suitable for this purpose [42]. Although EDTA has been shown to reduce CP oxidase activity in plasma samples, a high correlation between serum CP oxidase activity and EDTA-treated plasma samples has been shown, suggesting that a simple correction for EDTA inhibition of CP activity would be sufficient to obtain reliable values [43], no conversion factor has been developed. The use of this would enable standardization of the values of CP concentrations obtained in tests carried out using different methods and enable their comparison.

The concentration and/or activity of CP may depend on the *ATP7B* genotype. In patients with the p.R778L homozygous genotype, they had significantly lower serum CP levels (2.3 ± 0.5 mg/dL) than patients without R778L (6.2 ± 4.8 mg/dL) (*p* < 0.05) [44]. Similarly, patients homozygous for the p.H1069Q mutation had lower ceruloplasmin concentrations than heterozygotes with this mutation in one allele and homozygotes without this mutation in any *ATP7B* allele. Other studies have shown that nonsense mutations in the *ATP7B* gene associated with the formation of a truncated form of the protein are also associated with very low serum CP levels and early onset WND [45].

Heterozygous individuals with a mutation in one *ATP7B* allele usually have slightly reduced CP levels compared to healthy individuals [46].

The immunoassays used so far to determine the concentration of CP in the serum are based on indirect methods and may overestimate the amount of CP due to the presence of an apoprotein. The method of anion exchange chromatography with mass spectrometry with inductively coupled plasma with a triple quadrupole allows for the quantification of CP with a limit of detection of 0.1 mg/L and a limit of quantification of 0.4 mg/L [47]. The measurement of non-ceruloplasmin-bound copper (non-CP copper, NCC) with this device has also been investigated, and it has been noted that the ultrafiltration method for quantifying NCC is overestimated in the measurement due to the removal of copper from ceruloplasmin CP [47]. This test can provide a more accurate understanding of NCC in patients with WND.

Lopez-Avila et al. [48] presented an analytical method for the determination of CP in human serum by size exclusion (SEC) coupled with inductively coupled plasma mass spectrometry (ICP-MS). Unfortunately, this method has not been adequately tested in patients with WND. It has shown poor performance due to inadequately wide peaks and poor peak resolution [48]. This seems to be an inherent problem when using SEC separation to study intact metalloproteins, where even proteins with a significant difference in mass are not well separated because the cross-sectional size in solution is not different enough to allow separation [49,50]. Therefore, another work tested strong anion-exchange (SAX) chromatography, with initial studies focusing on gradient elution conditions, peak identification, and column washing procedure to maintain column performance after analysis of real samples [51].

Chromatographic peaks were identified by matching the retention time to known protein standards performed in various gradient elution programs to ensure no co-elution of metalloproteins. In addition, protein standards were added to real serum samples to confirm retention times.

Elemental speciation is increasingly used in various biomedical fields in a clinical or epidemiological context [52,53]. The combination of high performance liquid chromatography (HPLC) and tandem ICP-MS (ICP-MS/MS) may be the optimal tool for speciation studies [54,55]. New knowledge on the distribution and metabolism of Cu in WND may lead to improved diagnostic methods [56,57], new early screening strategies, and better treatment monitoring. The designed method should be tested on a larger cohort. The conducted study showed that it is applicable in WND studies.

It also appears to be a promising approach to study other Cu deficiency states that may develop in other disease states, such as malnutrition or malabsorption. Further stages of the implementation of the designed SAX-ICP-MS/MS method will be to improve the accuracy of CP quantification by using isotope dilution mass spectroscopy (IDMS) approaches [51,58].

#### 3.1.2. Serum Total Copper

The most sensitive and reliable method for the analysis of total copper concentration is atomic absorption spectroscopy. This test allows the assessment of total copper concentration in the serum. It has been noted that in patients with severe liver damage, serum copper concentrations may be within the normal range despite reduced serum CP levels. In patients with acute liver failure due to WND, serum copper may be markedly elevated due to the release of metal from tissue stores. A normal or elevated serum copper concentration with a concomitant decrease in serum CP indicates an increase in the concentration of non-CP copper in the blood [34,59,60,61].

The reduced total copper in the serum (below 62 μg/mL) have the greatest diagnostic value in the population of asymptomatic patients. In this case, the cut-off value is close to the previously established one (60 μg/mL) [14,62]. Total copper of <11 μmol/L correctly distinguished 96.4% of patients with WND from 86.7% of patients without WND, giving an overall accuracy of 89.2%. However, patients with WND and severe liver damage may have normal serum copper and some heterozygous WND carriers show reduced copperconcentration [63,64]. The normal serum copper in patients with low CP values indicates an increase in the fraction of NCC in the serum, which may suggest WND.

#### 3.1.3. Non-Ceruloplasmin-Bound Copper

Serum copper consists of two distinct pools. The main one (70–95%), considered non-reactive and therefore non-toxic, is associated with CP [65]. The remaining circulating copper (5–30%) is loosely bound mainly to albumin and to a lesser extent to alpha-2-macroglobulin, which are low molecular weight compounds (e.g., histidine). This labile pool used to be called “free copper” but with the development of new analytical methods now, the old term has been replaced with NCC [66,67]. Many different mathematical formulas have been proposed to calculate the fraction of free copper. According to the American Association of Liver Disease and European Association for the Study of the Liver guidelines, NCC may be estimated by subtracting the CP-Cu concentration from the total serum copper concentration (calculated NCC [cNCC]) [17,68]. 

Calculations are made using the following formula [35]:

“Free Copper” = Total copper (µmol/L) − 0.047 × CP (mg/L) (copper; serum copper concentration; CP; serum CP concentration)

This is a simplified formula. The factor of 0.047 is based on the knowledge that each CP molecule binds 0.3% of the copper, plus the knowledge of the copper atomic mass:

CP (mg/L) × 0.3/100 = bound copper (mg/L)

CP (mg/L) × 0.3/100 × 1000 = bound copper (µg/L)

Given that the atomic mass of copper is 63.6 g:

CP (mg/L) × 0.3/100 × 1000/63.6 = bound copper (µmol/L)

CP (mg/L) × 0.047 = bound copper (µmol/L)

It should be noted, however, that the results of the above calculations should be unreliable if the serum CP concentration is below 6 mg/dL [69].

In healthy individuals, the serum free copper concentration is usually less than 10% of the total copper concentration. In patients with WND, the NCC copper values exceed 30–50% of the total copper content. Serum free copper concentration was proposed as a diagnostic parameter of WND. In most untreated patients, it is elevated above 25 µg/dL (250 µg/L) (normal ≤15 µg/dL or ≤150 µg/L). NCC may be elevated in acute hepatic failure of any etiology and may be elevated in chronic cholestasis as well as in copper intoxication due to overconsumption or intoxication [39].

The main problem with the use of NCC in the diagnosis of WND is the correctness of both serum copper and CP measurement methods. If the serum copper measurement is inaccurate, or if the serum CP method overestimates the holoCP, the estimated non-CP copper concentration may be negative, and, for this reason, alternative parameters have been proposed. These include NCC percentage (NCC%), copper to CP ratio (CCR), and corrected copper [70].

Plasma-free copper values were calculated using the following formulas:

− NCC in molar units (μmol/L) = total copper (μmol/L) − [47 * CP (g/L)]
− NCC in mass units (μg/L) = total copper (μg/L) − CP-bound copper (μg/L)
− NCC% (%) = [NCC (μmol/L)/total copper (μmol/L)] * 100
− CCR (μmol/g) = [total copper (μmol/L) * 0.132]/CP (g/L)

NCC% is also affected by negative values that can be obtained in calculations based on total copper and CP concentrations, while CCR and corrected copper have the advantage of overcoming negative values. However, their clinical utility in WND is undetermined. European experts have estimated that the calculated NCC values are of greater value in monitoring the effects of treatment than diagnosing WND [71]. An NCC concentration of ≥5 µg/dL (≥50 µg/L) may indicate systemic copper depletion, which may occur in some patients on long-term treatment [68].

#### 3.1.4. Exchangeable Copper (CuEXC)

The new methods have been developed in order to directly calculate the NCC independently of the CP tests. One such method is the exchangeable copper test (CuEXC). This test uses a specific chelating agent to measure copper “exchanged” or mobilized from non-CP proteins and peptides. CuEXC corresponds to the labile fraction of copper complexed with albumin and, to a lesser extent, with α2M, amino acids, free Cu, and possibly with CP (type 1 Cu atoms) [72]. It is readily exchanged in the presence of chelating agents with high affinity for copper, such as EDTA [73].

Selective isolation of CuEXC from serum has previously been performed using extraction methods that were susceptible to copper contamination. These methods were based on the use of histidine, Chelex-100, and sodium diethyldithiocarbamate [74]. Balkhi et al. [75] proposed a CuEXC extraction method using ethylenediaminetetraacetic acid (EDTA), which is a copper chelator, in combination with a further ultrafiltration procedure preceding the determination of copper by atomic absorption. This method was validated for repeatability and long-term stability, and reference values of CuEXC were established in presumably healthy individuals (0.62–1.15 μmol/L Cu) [75], which were later used in the diagnosis of WND [74].

The new parameter, relative exchange copper (REC), reflecting the ratio of CuEXC to total serum copper, has been shown to be an accurate tool for the diagnosis of WND. In France, exchangeable copper-copper EXC (%NCC) and REC have been evaluated as promising tools for WND diagnosis and treatment monitoring. REC values > 18% have been found to have a sensitivity and specificity of 100% for diagnosis and family screening [76,77]. In presymptomatic, hepatic and extrahepatic presentations of the disease, the REC value was >15%. Increased CuEXC values were associated with extrahepatic symptoms. CuEXC values higher than 2.08 μmol/l allowed to distinguish the form of WND associated with the presence of hepatic and extrahepatic symptoms. These observations indicate that there may be a threshold concentration of CuEXC above which organs such as the brain and eyes (and possibly others such as kidneys and heart) may be affected by copper overload.

CuEXC may be a valuable tool for monitoring treated patients. In one study, CuEXC was normal only in a pre-symptomatic patient. Uncooperative patients with treatment failures based on transaminase elevations > 3 upper limit of normal (ULN) had elevated CuEXC values that were similar to those reported in new symptomatic patients. In contrast, normal or low CuEXC values were reported in compliant patients. Low CuEXC values in patients during treatment may indicate excessive copper depletion. Further studies are needed to assess the usefulness of the CuEXC test in monitoring treatment effects. Observations so far indicate that the CuEXC may be an alternative to the urinary copper excretion in monitoring the effectiveness of treatment [78].

The limitation that reduces the use of CuEXC is specific pre-analytical requirements: the blood sample must be quickly (within 30 min after collection) centrifuged, and the supernatant must be taken and frozen until the measurement, which must be performed within 7 days. Very few laboratories currently perform this test [79].

A recent Cu-speciation ICP-MS study [80], which was focused on the determination of Cu-CP value with the use of Cu-EDTA species-unspecific calibration strategy, showed an overestimation of CuEXC obtained with the conventional EDTA/ ultrafiltration procedure. This was explained by removing Cu by EDTA from Cu-CP. The ability of Cu speciation as an alternative approach to the current standard CP nephelometric method was shown in this study. However, Cu-species-specific calibration and/or protein recoveries were not yet investigated. The authors noted the need for accurate and metrological quantification strategies based on isotope dilution to validate routine analytic methods for Cu-species.

Recently, Quarles et al. [81] presented a direct and fast liquid chromatography inductively coupled plasma mass spectrometry (LC-ICP-MS) method for CuEXC testing. However, validation with a spike recovery experiment on real samples was not possible due the lack of well-characterized standards and the inability of the method to report individual Cu species (e.g., Cu-ALB).

Despite the great potential of linker separation methods by ICP-MS, Cu-specific detection in CuEXC studies, protein-targeted quantification methods for albumin-bound copper (Cu-ALB; main component of CuEXC), and Cu-CP are still rare, and no standardized reference methods have been developed.

Isotopic dilution mass spectrometry (IDMS) methodologies are needed for accurate quantification of protein species [82,83] as they can be invaluable as a basis for validating routine clinical methods and characterizing reference materials for method validation.

A new speciation approach has been described for the determination of the major Cu species in human serum (Cu-ALB and Cu-CP). It is based on the use of a relative peak area quantification strategy combined with determination of total Cu [80].

Evaluation of the accuracy of the Cu protein quantification method was performed by comparison with IDMS’s exact element and protein targeting strategies. Three commercially available human serum materials (two frozen LGC8211 and ERM^®^-DA250a sera and lyophilized Seronorm™ Human) with varying amounts of Cu species were used to validate the method, and degrees of measurement uncertainty were calculated. The validated approach was then applied to healthy and WND samples. In addition, it was used to investigate the potential bias of the classical EDTA/ultrafiltration method used for the determination of CuEXC.

#### 3.1.5. Albumin Copper (Cu-ALB) 

A custom protein quantification method was developed and characterized in terms of accuracy and uncertainty for the determination of serum Cu protein content, relevant for WND CuEXC analysis. It uses anion-exchange HPLC with an ICP-MS linker to determine the concentration of Cu bound to the protein fraction based on the relative peak area distribution and total Cu concentration in the sample. The high throughput of this method makes it attractive for screening Cu proteins in a clinical trial where large numbers of samples need to be analyzed within their stability window. Measurements of Cu-protein recovery determined from analysis of the same serum materials using species-specific isotope dilution mass-spectrometry (SS HPLC-ICP-IDMS) on Cu-ALB resulted in recovery values ranging from 102 ± 8 to 145 ± 12% for serum Cu-ALB concentrations between 455 and 112 μg/kg Cu. The relative expanded measurement uncertainty obtained for such concentrations of Cu in the serum with the newly proposed relative quantitative approach ranged from 5.7 to 10.1%. The relatively low limits of quantification (LOQ) for protein-bound Cu using this method allowed us to distinguish between the healthy individuals and the WND patients in terms of Cu-ALB concentrations. In addition, using this methodology for the first time, the underestimation of CuEXC by the classical EDTA/ultrafiltration method was demonstrated. The methodology developed in this work will be valuable in assessing quality control and monitoring WND drugs [80].

#### 3.1.6. New Alexion Test

A new method has recently been developed to assess copper metabolism during treatment with ALXN1840 (bis-choline tetrathiomolybdate [TTM]). This is an experimental copper binder that mobilizes copper in blood and tissues, and has shown a significant NCC-lowering effect in a phase 2.0 study. The mechanism of action of ALXN1840 is unique. It forms a neutral tetranary TTM-albumin-copper complex (TPC) that does not contribute to the exchangeable copper pool. To calculate the NCC in patients treated with ALXN1840, TPC would need to be measured and subtracted from the NCC9 fraction, and, therefore, a direct test is needed.

The assay directly quantifies many types of copper from human plasma using a combination of steps: (i) CP immunocapture using magnetic beads coated with a monoclonal antibody; (ii) chelation; and (iii) filtered inductively coupled plasma mass spectrometry (ICP-MS). These combined sequential processes enable the bioanalysis of CP and various types of copper, including CP-Cu, directly measured non-CP-bound Cu (dNCC) and labile bound copper (LBC), from human plasma. Validation experiments confirmed that this new assay directly measuring CP-Cu, NCC, LBC, and CP meets the established criteria of precision, accuracy, selectivity, and stability [84].

Data also support existing evidence suggesting that the ratio of CP-Cu:CP can be lower than the theoretical value of six in healthy people and in those with WND, and that calculation of NCC using an assumption of six atoms of copper per CP molecule is not accurate [85].

This assay could potentially be used for efficacy assessment of ALXN1840 in clinical trials and may have broader utility in diagnosis and treatment monitoring in patients with WND.

#### 3.1.7. Urinary Copper Excretion (UCE)

WND is characterized by increased urinary copper excretion, sometimes exceeding 100 µg/day, with the upper limit of normal being 50 µg/day. In untreated patients with WND, urinary copper excretion (UCE), which is a reflection of serum NCC, is elevated, and therefore measurement of copper in a 24-h urine collection is a common test for the diagnosis of WND. Urinary copper excretion greater than 100 µg/24 h is accepted as diagnostic for WND [86]. However, urinary copper excretion may be less than 40 µg/24 h at WND onset in 16–23% of patients, especially in children and asymptomatic siblings [25,87,88]. Assessing 24-h urinary copper levels, although one of the common approaches for the screening of families of patients with WND may result in misdiagnosis as 24 h urinary copper levels increase in other liver diseases and slightly elevate in carriers [89,90].

Similar to total serum copper, in several conditions, such as autoimmune hepatitis, cholestasis syndromes, and acute liver failure, urinary copper is elevated as a compensatory mechanism to altered biliary excretion. In the Cochrane database systematic review, a cut-off of 0.64 to 1.6 µmol/24 h (40–100 µg/24 h) used in the Leipzig criteria achieved a variable sensitivity of 50–80%, with a specificity of 75.6–98.3% [29].

Patients with certain chronic liver diseases, including autoimmune hepatitis, may have basal 24 h copper excretion in the range of 100–200 µg/24 h (1.6–3.2 µmol/24 h) [89]. In one study, 5 of 54 patients with chronic active liver disease had urinary copper excretion above 100 µg/24 h; overlap has also been reported in children with autoimmune hepatitis [91].

Reference ranges for normal 24 h copper excretion vary between laboratories.

In symptomatic patients, values of 1.2 to 2 times the upper limit of normal (ULN, set at 100 µg/24 h) are recorded, which may indicate possible WND. Unfortunately, method-dependent cutoffs provide a low negative predictive value (NPV) for this test, with up to 25% of people with confirmed WND (particularly children) having urinary copper levels near normal at disease onset [33]. In addition, heterozygous WND carriers may have intermediate values. To solve this problem, it has been proposed to lower reference levels to <40 µg/24 h (0.6 µmol/24 h) in the diagnosis of pediatric and asymptomatic patients [68].

Copper concentration is determined in a 24-h urine collection.

Patients must follow specific specimen collection instructions in order for their urine copper excretion test results to be reliable. Unfortunately, these results may be affected by sampling errors. In order to obtain accurate test results, it is recommended, among other things, to use disposable polyethylene urinals for collecting urine, which prevent copper contamination of the urine sample. Urine copper measurements can provide useful diagnostic information as long as copper does not contaminate collection vessels and urine collection is complete.

The urinary copper excretion and serum copper concentrations are useful for monitoring patient compliance and adherence to chelation therapy. 24 h urinary copper excretion is believed to be more reliable than NCC for confirming adherence and treatment success [86].

In general, patients taking D-penicillamine or trientine are expected to have urinary copper excretion in the range of 200–500 µg/24 h, although if 24 h urinary copper excretion is measured 48 h after the end of treatment, it is expected that values will be close to normal (<50 µg/24 h) [17]. Levels < 75 µg/24 h indicate the effectiveness of treatment in people using zinc therapy [12]. Higher excretion rates are usually observed in the first months after starting chelation therapy for further stabilization in the long term. New tools, in particular the direct determination of NCC using the copperEXC method, will facilitate patient compliance and treatment monitoring, but their availability is still very limited.

A popular modality of UCE is D-penicillamine test. The measurement of copper in the urine after stimulation with 1000 mg of D-penicillamine is a test proposed for diagnostics in children, but not in adults; however, the data are ambiguous, even in children [17].

This test has only been standardized in a pediatric population of 57, which was initially given 500 mg of oral D-penicillamine and again 12 h later during a 24 h urine collection, regardless of body weight. Compared to other liver diseases, including autoimmune hepatitis, primary sclerosing cholangitis, and acute liver failure, there was a clear differentiation with excretion of >1600 µg copper/24 h (>25 µmol/24 h). A recent re-evaluation of the penicillamine challenge test in children showed its usefulness in diagnosing WND in patients with active liver disease (sensitivity 92%), but not sufficient to exclude the diagnosis in asymptomatic siblings (sensitivity only 46%) [24]. Others have found that the predictive cut-off value of 25 µmol/24 h should be >100% [92,93]. This test has been used in adults, but many of the reported results of this test in adults have used different doses and timings of D-penicillamine administration [87,89,90].

#### 3.1.8. Test with Radioactive Copper

The radioactive copper test is based on the intravenous administration of the copper isotope copper-64 in the form of an isotonic solution of copper hydrochloride. After two hours, blood is drawn, and radioactivity is determined in 2 mL of serum. Radioactivity is measured in a scintillation counter. The result is reported as the ratio of the radioactivity at 24 h to the radioactivity at 2 h after isotope administration and as the radioactivity of the sample measured at 48 h relative to the measured value at 2 h. The results obtained should be corrected for the physical decrease in the radioactivity of copper, taking into account the half-life of 12.9 h. In healthy individuals, serum radioactivity drops sharply in the first three hours after administration of the isotope. This is the result of the gradual absorption of copper by the liver. Since copper binds to CP under physiological conditions, a secondary increase in serum radioactivity is observed after about 12 h. Healthy individuals show values in the range of 1.04–1.50 if the radioactivity is measured after 24 h (64copper 24/2) and 0.85–2.03 if the radioactivity is measured after 48 h (64copper 48/2) [94,95].

Patients with WND showed significantly lower copper ratios compared to healthy individuals and carriers of one *ATP7B* mutation with a sensitivity of 98.6% for 48 h/2 h 64copper ratio [95,96]. The radioactive copper test is very useful when the results of routine biochemical tests and clinical observations do not allow for unequivocal confirmation or exclusion of the disease. The result of this test sometimes determines the diagnosis.

In WND patients with normal serum CP, radiocopper incorporation into this protein is significantly reduced compared to healthy individuals and most heterozygotes. The lack of incorporation of copper into the plasma protein in hepatocytes occurs in all homozygotes with this disease. 

#### 3.1.9. Mass Spectrometry in Dried Blood Spot (DBS)

An alternative method of diagnosing WND is the quantification of the ATP7B peptide by immunoaffinity enrichment mass spectrometry in dried blood spot (DBS). A pilot study was conducted on thirteen patients with WND; the assay precision was <10% CV and the protein was stable for one week in DBS at room temperature [97].

#### 3.1.10. Intrahepatic Copper Quantification

In patients with suspected WND, the determination of copper content in a liver sample may be helpful in diagnosing the disease. In healthy people, these values are less than 50 µg of copper/g of dry tissue [98]. In 80% of patients, values even above 250 µg copper/g dry tissue are found. Copper content in the liver parenchyma >250 µg/g dry weight provides critical diagnostic information, and further diagnostic testing is indicated in patients with intermediate copper levels (70–250 µg/g dry weight), especially if there is active liver disease or other symptoms of WND.

Copper dry matter is increased in 80% to 96% of patients but may be false negative due to extensive fibrosis and false positive in chronic cholestasis. Markedly elevated liver copper concentration may also be found in idiopathic copper poisoning syndromes such as Indian childhood cirrhosis [99]. In long-term cholestatic disorders, the copper content in the liver may also be increased above this level.

However, this threshold value has been criticized as being too high and based on relatively few cases: based on an analysis of 114 genetically confirmed cases, it has been suggested that a threshold value of 70 μg/g dry weight dramatically increases sensitivity, albeit with some loss of specificity. In another study, all patients had a liver copper content of at least 95 μg/g dry weight, and 92% of patients had liver parenchymal copper concentrations > 250 μg/g dry weight [100]. Normal liver copper content (<40–50 μg/g dry weight) almost always excludes WND [100].

The main problem with the liver copper test is that the deposition of copper in the liver structures depends on the severity of the disease. Thus, the quantification of intrahepatic copper is not always reliable. In the later stages of WND, the distribution of copper in the liver is often heterogeneous, so the liver sample is not usually representative and results of liver copper concentrations are generally underestimated [101]. In extreme cases, nodules devoid of histochemically detectable copper are found next to cirrhotic nodules with a large amount of copper. Therefore, the concentration may be incorrectly estimated due to sampling error. In a pediatric study, sampling error was common enough to render the test useless in patients with cirrhosis and clinically evident WND [102].

Sometimes, despite the presence of liver damage, the values of copper content in dry tissue are within normal limits. This may be due to the heterogeneous distribution of copper deposits in the liver tissue. Various studies have shown up to 500-fold differences in copper values in different parts of the liver tissue [17].

In general, measurement accuracy improves with appropriate sample size: at least 1–2 cm of biopsy core length should be analyzed [103].

Most patients refuse a liver copper test because it is invasive. Clinical practice guidelines recommend intrahepatic copper quantification only when other evidence is insufficient to make a diagnosis, or if other liver pathology is suspected in addition to WND.

### 3.2. Copper Monitoring in Special Situations in WND

#### 3.2.1. Neonatal Screening

Due to the relative frequency of WND and the availability of rational therapy, it would be advisable to include WND in the list of diseases examined as part of newborn screening. Holo-CP, serum copper, and UCE methods have limited predictive value in children. An alternative to routine biochemical tests may be the quantification of ATP7B protein in DBS, as mentioned earlier [46]. Finally, next generation sequencing (NGS) has reduced the cost and time required for enhanced efficacy, placing the technology within reach of clinical routine [104].

#### 3.2.2. Presymptomatic Diagnostics

Of all the diagnostic tests used, apart from genetics, it has been found that serum CP and serum total copper may be the best predictors of disease in asymptomatic individuals, compared to other basic parameters such as 24 h urinary copper excretion, copper content in the liver, or the presence of Kayser-Fleischer (KF) rings.

ROC analyses first showed that serum CP, with a cut-off value of 11.5 mg/dL, had an area under the curve (AUC) of 0.99 (95% CI 0.97–1), with a specificity of 1 and a sensitivity of 0.97. At this cutoff, the PPV was 1 and the NPV was 0.977, compared to PPV = 0.92 and NPV = 0.975 for the 15 mg/dL threshold. Second, serum copper, above 62 µg/dL, showed the highest specificity, 1, with a sensitivity of 0.971, also showing an AUC of 0.99 (95% CI 0.97–1). In this case, PPV = 1 while NPV = 0.96. Copper in urine collected over a 24 h period was also included in the analyses, revealing that a cut-off value of 21.5 µg/24 h, with a specificity of 1 and a sensitivity of 0.909, which was required to distinguish cases from asymptomatic individuals, representing an AUC of 0.993 (95% CI 0.981–1) [26,28,36].

#### 3.2.3. Diagnosis in Children

There have been few studies evaluating the diagnostic accuracy of serum CP in children with WND [28,30,31,105]. Serum CP is physiologically very low in early infancy until the age of 6 months, peaking at higher than adult levels in early childhood (approximately 300–500 mg/L) and then returning to the adult range. Therefore, it is important to establish age-appropriate reference values for serum CP concentrations in healthy children and to evaluate the diagnostic criteria for serum CP levels for WND in early childhood [26].

Some authors believed that the norms should also take into account the gender of children. In one study, serum CP levels were higher in boys than in girls among healthy children (*p* < 0.01) and among patients with WND (*p* < 0.05) [26]. A previous study showed that serum CP did not differ by gender, with the exception of higher levels in girls during puberty due to estrogenic stimulation [106,107]. Another study provided very different results in both healthy children and asymptomatic WND patients based on a large cohort of pediatric WND patients and healthy controls. Therefore, the gender difference in serum CP should probably be taken into account when diagnosing WND in childhood, but further research is needed [11,108].

Earlier reports also indicated that serum CP is not suitable for newborn screening. However, the serum CP level may be one of the sensitive biomarkers for the diagnosis of WND in children older than 6 months [11,109].

#### 3.2.4. Copper after Liver Transplantation

Liver transplantation has been reported to normalize copper metabolism in recipients [110,111].

Cadaveric liver transplantation has been reported to normalize copper metabolism in recipients [111,112]. Since the scarcity of cadaveric organs is a serious problem in many countries, LRLT is also indicated for this disease [113,114,115]. After living-associated liver transplantation (LRLT), all recipients had normal serum CP levels and a marked decrease in urinary copper excretion. All donors had CP values within the normal range; also the recipients had normal CP levels after transplantation. In addition to normalization of laboratory parameters of copper metabolism, 5/8 patients with Kayser–Fleischer rings had complete resolution, and the remaining three showed improvement after transplantation. Despite these results, it should be remembered that about 10% of WND heterozygotes may have a reduced level of CP, so they may not be suitable as donors [110]. 

However, Asonuma et al. reported that LRLTs from heterozygous donors who carry mutations in one ATP7B allele can resolve clinical symptoms and improve parameters of copper metabolism [116].

**Table 1:** Major biochemical tests for Wilson’s disease.

Method	Measurement	Reference Range	Cut-off Values for Wilson’s Disease	Most Important Limitations
**Ceruloplasmin enzyme assay**	Concentration of holo-ceruloplasmin in serum based on enzymatic activity colorimetric method	25–45 mg/dL	<20 mg/dL [32]	False negative results during inflammation, hepatitis, pregnancy, in patients taking oral contraceptives; at low values, it is necessary to differentiate with aceruloplasminemia
**Ceruloplasmin nephelometric assay**	Concentration of apo- and holo-ceruloplasmin in serum. Test based on monoclonal antibodies	25–45 mg/dL	<20 mg/dL [32]	As above; additionally overestimation of the CP due to apoprotein detection
**Serum total copper**	Serum copper concentration by atomic absorption spectroscopy	70–155 μg/dL	<60 µg/mL [14,62]	False negative results in case of severe liver damage
**Non ceruloplasmin bound copper (NCC)**	Estimation by subtraction the CP-Cu concentration from the total serum copper concentration (calculated NCC [cNCC])	≤15 µg/dL or ≤150 µg/L	>25 µg/dL or 250 µg/L [39]	cNCC results may have aberrant negative value if CP is low
**Exchangeable copper (CuEXC)**	Serum copper “exchanged” or mobilized from non-CP proteins and peptides measured by chelating or extraction methods or chromatographs	4.1–7.1 μg/dL reference intervals are age-specific	>7.1 µg/dL [75]	The blood sample must be within 30 min after collection centrifuged and stored, measurement, must be performed within 7 days
**Albumin-copper**	Concentration of serum protein bound copper by chromatography	122–455 μg/kg body weight	Not established	Under development
**Urinary copper excretion**	Copper in a 24-h urine collection	<50 µg/24 h	>100 µg/24 h [86]	False positive results in patients with autoimmune hepatitis, cholestasis syndromes and acute liver failure, false negative results in children and asymptomatic patients, frequent sampling errors
**Test with radioactive copper**	Intravenous administration of the copper isotope copper-64, measurement of blood samples radioactivity at 2, 24 and 48 h thereafter.	24 h/2 h Cu^64^ ratios:1.04–1.50 48 h/2 h Cu^64^ ratios:0.85–2.03	24 h/2 h Cu^64^ ratios: 0.14; 48 h/2 h Cu^64^ ratios: 0.12 [96]	Limited availability, long duration of the test (48 h)
**Copper concentration in the liver**	Copper concentration in liver sample	40–50 µg/g dry weight	>250 µg/g dry weight [100]	Invasive method- liver biopsy necessary, false negative results possible as copper deposits in the liver are distributed unevenly, false positive results in cholestatic liver diseases

## 4. Conclusions

There are many diagnostic and monitoring tools available for patients with WND. While many patients are diagnosed and adequately assessed with currently available methods, diagnosis and monitoring have proven more difficult for other patients who are stuck with diagnostic uncertainty characterized by borderline CP levels, inconclusive genetic findings, and unclear clinical phenotypes. The patient’s prognosis is based on early diagnosis and appropriate long-term therapy. Delaying diagnosis can be catastrophic. Technological progress and characterization of new diagnostic parameters, including those related to copper metabolism, may provide confidence in the more accurate diagnosis of WND in the future [16].

## Data Availability

Data sharing not applicable; no new data were created or analyzed in this study. Data sharing is not applicable to this article.
